# Children With Sydenham Chorea and Psychiatric Disorders Had Variable Long‐Term Outcomes and Required Multidisciplinary Management

**DOI:** 10.1111/apa.17590

**Published:** 2025-01-22

**Authors:** Nadine Mushet, Michael Morton, Helen Minnis, Christopher Gillberg

**Affiliations:** ^1^ Children and Young People's Services Cumbria, Northumberland, Tyne & Wear NHS Trust Newcastle UK; ^2^ Department of Health and Wellbeing University of Glasgow Glasgow UK; ^3^ Gillberg Neuropsychiatry Centre, Institute of Neuroscience and Physiology, Sahlgrenska Academy Gothenburg University Gothenburg Sweden

**Keywords:** auto‐immune neuropsychiatric disorders, movement disorder in childhood, post‐streptococcal disorder, rheumatic fever, Sydenham chorea

## Abstract

**Aim:**

Sydenham chorea (SC) is a globally significant, post‐streptococcal, childhood neuropsychiatric condition that is rare in western Europe. This retrospective single‐centre study focused on children with neuropsychiatric features of SC.

**Methods:**

Participants were recruited from neuropsychiatry referrals to a regional paediatric neurology department in Glasgow, Scotland, from 2009 to 2012. Interviews established the course of SC and the children's medical and family histories. Semi‐structured psychiatric interviews explored current and past episodes of psychopathology.

**Results:**

We studied 12 children (seven girls) with a mean age of 13 (range 10–15) years, and an average of six (range 4–10) years after their SC symptoms began. Before they displayed symptoms, seven children had been suspected or diagnosed neurodevelopmental problems and four had separation anxiety. Their physical symptoms were often debilitating. Psychopathology was most severe during their first episode and SC diagnoses were sometimes delayed. Educational problems were frequent. Multiple psychiatric conditions were diagnosed in 11 children and anxiety and attention deficit hyperactivity disorder were the most common. Relapses occurred in nine cases. Additional services that were accessed included cardiology, rheumatology, physiotherapy, occupational therapy and speech therapy. Medication included prophylactic penicillin and symptomatic treatment.

**Conclusion:**

Children with SC and psychiatric disorders had variable long‐term outcomes and required multidisciplinary management.

**Abbreviations**
ADHD: attention deficit hyperactive disorder
ASD: autism spectrum disorder
DSM‐IV: Diagnostic and Statistical Manual of Mental Disorders, Fourth Edition
K‐SADS‐PL: Kiddie Schedule for Affective Disorders and Schizophrenia for School‐age Children—Present and Lifetime Version
PANDAS: paediatric autoimmune neuropsychiatric disorders associated with streptococcal infection
SC: Sydenham chorea

**Summary**

- Sydenham SC (SC) is a globally significant, post‐streptococcal, childhood neuropsychiatric condition, but it is rarely seen in Western Europe.
- This Scottish study carried out retrospective interviews with the families of 12 children to identify the timelines of their psychiatric and physical symptoms.
- Key findings of the study were that children with SC and psychiatric disorders had variable long‐term outcomes and required multidisciplinary management.

## 1 Introduction

Sydenham chorea (SC) is a post‐streptococcal, autoimmune, neuropsychiatric movement disorder and it is the most common global cause of chorea in childhood [1]. It is characterised by the purposeless, involuntary and non‐stereotypical movements of chorea. These typically occur after children aged 5–14 years are infected with group A streptococcus [2]. SC is a major criterion for diagnosing acute rheumatic fever and could be the only manifestation of rheumatic fever when a child first presents. This has implications for identifying other important potential sequelae, including cardiac complications [3]. Chorea in SC can be severely debilitating. It can cause problems with speech, swallowing, writing and walking and paralysis can occur in severe cases. Psychiatric comorbidities have been well established in SC, but the prevalence of psychiatric diagnoses associated with SC has varied [4]. Although the initial episode of SC usually resolves in 1 to 2 years, SC can become a chronic condition and is sometimes relapsing and remitting.

The global incidence of SC is thought to be closely related to the incidence of rheumatic fever. Both conditions have shown a marked decline in Europe, but remain important causes of morbidity in socially and economically disadvantaged populations in the developing world [5]. SC is rare in Western Europe. A UK and Ireland surveillance study from 2018 to 2020, reported an annual incidence rate in children aged 0–16 years of 0.16 per 100 000 [6]. This was similar to a study in Ireland, from 2006 to 2014, that showed an annual incidence rate of SC of 0.23 per 100 000 children under 14 years of age [7]. That study suggested that clinicians may have been less skilled in recognising and managing this disorder when they worked in areas where the incidence of rheumatic fever was low [7]. Diagnosing SC relies on a clinical finding of chorea and excludes alternative aetiologies. Serology is not always positive, as disease onset may be up to 8 months after an initial infection with group A streptococcus [8].

Little is known about the natural history of the neuropsychiatric problems associated with SC over time. Heritable vulnerability to SC has been reported [9], but there is limited information about the phenotypical features of children at increased risk of SC. There has been much interest in the occurrence of neuropsychiatric symptoms after streptococcal infections that were not associated with rheumatic fever or SC. This has led to the proposed diagnosis of paediatric autoimmune neuropsychiatric disorder associated with streptococcal infection (PANDAS) [10]. A paper by Swedo et al. discussed perspectives on the relationship between PANDAS and SC and described proposals for a new term, namely Paediatric Acute‐onset Neuropsychiatric Syndrome [11]. The difficulties with these concepts have been highlighted [12] and these conditions remain controversial, unlike SC.

This in‐depth Scottish study aimed to describe the symptoms and experiences of a cohort of children with SC, with associated psychiatric disorders, who were assessed at a regional paediatric neurology department. We believe it is the first UK study of SC and the onset of psychiatric symptoms and sequelae.

## 2 Methods

This study focused on children who were referred to the Neuropsychiatry Clinic at the Royal Hospital for Sick Children, Glasgow, Scotland after SC was diagnosed in the hospital's Regional Paediatric Neurology Department. Children referred to the Neuropsychiatry Clinic between 1 April 2009 and 31 March 2012, who were under 18 years of age at the time of the study, were eligible to take part. There were no other exclusion criteria. Referrals were identified from psychiatry records. SC was diagnosed by a paediatric neurologist, based on the child's clinical history and a neurological examination that confirmed the presence of chorea. Potential cardiac, psychiatric and rheumatological complications were also considered. Antistreptolysin‐O tests and echocardiography were part of the routine work‐up, but negative findings did not preclude a diagnosis. Further investigation was undertaken, as indicated, to exclude alternative diagnoses. These were routinely discussed at a weekly video‐diagnostic meeting. Around half of the children with SC who were seen by the paediatric neurologists had psychiatric symptoms that led to a referral to the Neuropsychiatry Clinic. An audit confirmed that all the assessments met the standard set by the Regional Paediatric Neurology Department.

During the period studied, 19 children were referred to the Neuropsychiatry Clinic with confirmed diagnoses of SC, but three were excluded because they were not under 18 *changed to be consistent with other text* years of age. The families of three boys and one girl did not respond after two reminders were sent by post. Data are reported on 12 children (75%) from the West of Scotland Region, which comprised around 555 000 children under 18 years of age in 2016 [13].

The families were contacted by letter and invited to take part in research interviews. The research team then contacted them and offered them appointments. The parents and children were seen both together and separately.

The assessment included taking a standard medical and family history from the family. This was used to construct a detailed timeline of physical, developmental and psychiatric problems related to the onset of SC. The assessors also gathered information about the services the child used and the treatments they received. Subsequent presentations to services were recorded, along with when they were diagnosed and any subsequent relapses. The children's medical files were not accessed. Each child was video recorded during a brief neurological examination, which included screening for current chorea using the Universidade Federal de Minas Gerais Sydenham Chorea Rating Scale [14]. A child and parent semi‐structured psychiatric diagnostic assessment interview was conducted using the Kiddie Schedule for Affective Disorders and Schizophrenia for School‐age Children—Present and Lifetime Version (K‐SADS‐PL) [15]. The K‐SADS‐PL is validated for children under 18 years of age and was designed to explore current and most severe past episodes of psychopathology, according to Diagnostic and Statistical Manual of Mental Disorders, Fourth Edition (DSM‐IV) criteria [16]. It includes screening questions for key symptoms and supplementary diagnostic assessments for 20 psychiatric disorders. If a participant screened positive for a key symptom during our study, supplementary questions were asked about the remaining criteria for that specific disorder. If sufficient symptoms met the relevant thresholds, diagnoses were considered for the current presentation and/or past episodes.

The K‐SADS‐PL findings were obtained by three different interviewers: one performed eight interviews and two performed two interviews each. These findings were used to create timelines that described the development of behavioural, psychiatric and cognitive problems in relation to the onset of neurological symptoms and the timing of SC diagnoses. We collated the data for the DSM‐IV diagnoses at the time of the SC diagnosis, during the most severe episode and at the time of the interview. Two of the three interviewers had a certificate in administering the K‐SADS‐PL and they trained the third to use the instrument.

### 2.1 Ethics Statement

The study was approved by the West of Scotland Research Ethics Committee (number GN14NE219). All the parents and participants provided written, informed consent or assent, as applicable, at the start of the first interview.

## 3 Results

At the time of interview, the 12 children (seven girls) had a mean age of 13 (range 10–15) years. They were all Caucasian. The families had initially sought help from professionals, such as family doctors, helplines and emergency departments, because of the onset of physical symptoms. None had sought advice from psychiatric services. The children's mean age at the time of their SC diagnosis was nine (range 4–14) years and the interviews were conducted at a mean of six (range 4–10) years after the SC symptoms began.

### 3.1 Family Factors

First‐degree relatives with a history of rheumatic fever were found in three of the 12 families and nine reported psychiatric disorders in their family, with depression being the most common diagnosis. Four mothers reported that they currently had depression and three suggested that their child's SC affected their mood. Four described previous psychiatric disorders in first‐degree relatives and a further five in second‐degree relatives. Only one family reported no disorders in their relatives.

### 3.2 Problems Before SC Symptoms Appeared

Questions about the symptoms that were present before the onset of SC revealed developmental vulnerabilities and/or psychiatric symptoms in all 12 cases. An undiagnosed learning disability was identified in one boy and four boys and one girl had experienced motor and/or speech delays. Past neurological histories included a head injury that caused loss of consciousness, neonatal meningitis, longstanding severe migraines and uncomplicated absence epilepsy. The parents of one boy and three girls said they did not display psychiatric symptoms before their first presentation of SC, but they had subsequently developed behavioural symptoms. Two boys and two girls had displayed clingy and anxious behaviour, and it had been difficult to make them go to school or nursery. However, this had resolved over time. Two more feared attending school because they felt isolated and lonely. Another child was described as emotional following tonsillitis. One child had behavioural problems as an infant, 4 years before diagnosis, and another had mood swings. Two had been restless, but their periods of impulsivity and concentration difficulties did not meet the criteria for attention deficit hyperactive disorder (ADHD).

### 3.3 Symptoms During First SC Presentation

The parents of eight children recalled seeing some behavioural changes before the physical signs of movement disorder appeared. However, all the children had physical symptoms by the time their parents first sought help from professionals, such as family doctors, helplines and emergency departments. Physical symptoms were variable, but eight of the children were significantly impaired or disabled. There were eight children with disrupted gross motor skills, six had speech difficulties, six had joint pain, one had a rash and one showed signs of cardiac valve disease. Only five of the 12 sets of parents used the word chorea when describing motor symptoms. Three of the five described these as tics.

At their first presentation, two of the children had mobility problems and a parent described hypermobility in one child. Cardiac problems were identified in two boys and two girls. Two children had enuresis, one had pneumonia, and one had sleep difficulties. Sometimes, early physical signs of SC were poorly recalled. There were four children with a history of tonsillitis and one of these had undergone a tonsillectomy.

At the time of their SC diagnosis, six children required a wheelchair and six needed aids, such as adaptive cutlery, a commode or a walking‐frame. Sore throats were reported in two children, poor bladder control in two and loss of appetite in one. All received penicillin for SC and 11 continued prophylactic penicillin, but one could not tolerate this. Sodium valproate was prescribed for chorea in eight children. Analgesics were given for joint pain in seven cases, omeprazole was given for reflux in two cases and lisinopril was prescribed by cardiology for one child. None of the children received immunotherapy.

During the month leading up to their SC diagnosis, two children had not shown any psychiatric symptoms. The parents of eight children described anxiety symptoms, including six children with generalised and separation anxiety and two with just separation anxiety. Specific phobias were identified in two children, a social phobia in one and obsessional compulsive behaviour in another. Tics were noted in three children and two had attention and concentration difficulties. One child had depressive symptoms and another experienced panic attacks. Sleep problems affected two children. Hallucinations were experienced by two children and two had social communication difficulties. Parents described transient difficulties with aggression and disinhibition.

When the children were first seen in neurology, four presented with behavioural problems, disruptive emotional episodes and unusual behaviours, such as difficulties understanding instructions. Two children were still reporting hallucinations. In one case these were attributed to their medical condition and associated fatigue. Two boys and one girl reported tics, with one reporting restlessness that did not meet the criteria for ADHD. A child with symptoms that included tics, gait abnormality, sleep difficulties and separation anxiety was assessed for PANDAS.

### 3.4 Psychiatric Disorders and Relapses

At the time of their SC diagnoses 10 of the 12 children met the diagnostic criteria for at least one psychiatric disorder, according to the DSM‐IV. One girl and one boy did not. One boy and three girls had multiple diagnoses (Table 1). In five children, the first manifestation of psychopathology associated with SC was also the most severe past episode of a psychiatric disorder. The K‐SADS‐PL interview established that all had received at least one psychiatric diagnosis during their lifetime worst episode. Anxiety disorders were common, with generalised anxiety in six children and separation anxiety in eight. Three of the children with anxiety disorders also had obsessive‐compulsive disorder and three also had depression. ADHD was identified in seven cases, and all of these had anxiety disorders. Tic disorders were found in three children. Two had symptoms of autism spectrum disorder (ASD), but one child no longer met the criteria after the acute phase of SC. Auditory hallucinations were also limited to the acute phase of SC in one child. Over the course of the study period, 11 children met the criteria for more than one psychiatric diagnosis. The psychotropic drugs that were prescribed included anti‐psychotic medication for one child, antidepressants for two and carbamazepine, as mood stabiliser, for one. Melatonin was used for two children with sleep difficulties and clonidine for one child for tics.

**TABLE 1:** Number of children who met the thresholds for DSM‐IV diagnoses using the K‐SADS‐PL.

DSM‐IV psychiatric diagnosis	Children affected at time of their SC diagnosis	Children affected during their most severe psychiatric episode^a^	Children affected at the time of the research interview
Anxiety disorders			
Generalised anxiety	4	6	4
Separation anxiety	2	8	2
Obsessive compulsive disorder	—	3	—
Social phobia	2	2	5
Specific phobia	3	3	—
Panic disorder	3	4	3
Neurodevelopmental disorders			
Tic disorder	3	3	3
ADHD	5	7	5
Co‐morbid oppositional defiant disorder & ADHD^a^	—	(2)^a^	
ASD	1	2	1
Learning disability	1	1	1
Mood disorders			
Depression	—	3	—
Hypomania	1	2	1
Psychosis			
Psychosis	—	2	—

^a^ This was the lifetime worst episode, which was the most severe past episode of psychopathology.

Only three children had no SC relapses. Relapses with recurrent chorea and other physical and psychiatric symptoms were recalled in six cases. Joint pain accompanied relapses in three cases, with mobility problems in two. SC relapses were primarily characterised by psychiatric symptoms in three children. Symptoms that suggested diagnoses of ADHD, obsessive compulsive disorder, depression, separation anxiety, panic disorder, phobias and ASD were found during relapse episodes. The worst psychiatric disorder episode coincided with the first episode of SC in nine of the 12 children. The other three had more severe psychiatric symptoms during their first relapse. Most of those with ADHD recovered from symptoms during relapses, but anxiety disorders were the most likely to persist. One child had a persistent tic disorder, and another had a recurrent mood disorder. Six children had single relapses, but two girls and one boy had multiple relapses and multiple psychiatric diagnoses and two of these developed more persistent and severe presentations.

Three children had current subjective experiences of chorea and they, and two other children, had objective signs of chorea on examination. The parents of all of these rated their child's functional impairment from their chorea as minimal to moderate.

Cardiac reviews continued in three children and three had persistent enuresis. One child was attending local child and adolescent mental health services for pharmaceutical interventions and psychoeducation for tics. Two other children were attending liaison psychiatry clinics for pharmaceutical interventions, one for depressive episodes and one for generalised anxiety and panic.

### 3.5 Experience of Services

The children and their families said that they had contact with a range of services to address the changing physical and psychological needs arising from SC. Nine visited their family doctor in the first instance and four attended hospitals as emergencies, either following referrals or independently. During the first presentation, six were offered advice regarding possible viral infections without any follow up. Two children were followed up and their parents initially believed that they were being investigated for brain tumours.

Seven children were diagnosed with SC soon after the onset of symptoms, but five experienced delayed diagnosis, with an average of 10 months (range 3–24 months). Diagnoses of SC were made by neurologists in six cases and the rest were initially diagnosed by a paediatrician. Parents reported that difficulties reaching a diagnosis caused significant stress in addition to the child's distress.

All children received multi‐disciplinary support, with eight reviewed in cardiology clinics and five in rheumatology clinics. There were eight referrals to physiotherapy, six to occupational therapy, three to dietetics, three to speech and language therapy and one each to play therapy and an incontinence service. Referrals to neuropsychiatry were made soon after the child's diagnosis in 10 cases and two were referred three and 12 months later. Psychological interventions included individual and group work with the children, parental support and advice on education. Some required longer follow ups, particularly for anxiety or mood disorders, and ADHD presentations.

Physical, behavioural and cognitive problems in school were often reported and temporarily disrupted learning. New educational concerns were raised by the children, parents and teaching staff, and two children obtained support from an educational psychologist. More than half reported being bullied and this was associated with anxiety at school and reduced attendance.

## 4 Discussion

Little has been published about the progression of neuropsychiatric features in SC. Our study used semi‐structured interviews to provide accounts of the longer‐term course of SC in children referred to psychiatry professionals from a regional paediatric neurology service. It is important to note that SC could be particularly distressing for the family and the children from the onset of symptoms if psychiatric symptoms were an early feature. In most cases, reported signs of behavioural, psychiatric and cognitive problems emerged over a significant period and were related to the onset of physical symptoms. Some were present before the onset of SC, but others were noticed early in the development of SC, sometimes before the physical symptoms were recognised.

The K‐SADS‐PL provided us with the framework to carry out detailed explorations of the experiences of SC alongside potential psychiatric diagnoses. Our findings were in keeping with previous research. Psychiatric disorders were often time‐limited, but various prolonged neuropsychiatric sequelae and relapses had an impact on the children's functioning. Rates of current psychiatric morbidity and co‐morbidity appeared high in comparison to population norms. Around half of all the children diagnosed with SC in Glasgow were referred to neuropsychiatry services. Our findings suggest that at least 30% of the children who presented may have reached the criteria for a diagnosis of ADHD. This would have greatly exceeded the average 5% global prevalence for ADHD [17], but was in keeping with previous research on SC [18]. One American study that used the K‐SADS‐PL [19] found that a similar rate of ADHD, even before the onset of SC. Variations in reports of ADHD symptoms over time may reflect difficulties in using the K‐SADS‐PL retrospectively to distinguish ADHD symptoms from motor restlessness secondary to active chorea. Affective and anxiety disorders have been reported to be prominent in SC [20], while separation anxiety has been particularly associated with PANDAS [21]. One striking finding of our study was that two children reported hallucinations that were sufficient for a DSM‐IV diagnosis of psychosis.

Medical staff did not always recognise that the children required paediatric reviews at the time of their first presentation, even though the emerging SC symptoms were causing anxiety. Stress might have been reduced if this delay in diagnosis had been avoided. Our findings suggest that there may have been a risk of misdiagnosing SC if the specific physical symptoms of SC were not often recalled as a prominent feature of the acute illness. This could arise during the initial assessment and in re‐presentations with predominantly psychiatric symptoms. Other studies have highlighted the cardiac difficulties that could be missed if clinicians did not recognise the rare presentation of chorea [22, 23]. This, in addition to delayed diagnosis, could have contributed to high rates of psychiatric symptoms occurring before SC diagnoses in our study being presented in this paper.

A possible predisposition to SC has been suggested by findings of developmental delays and pre‐existing neurological conditions, as well as descriptions of pre‐existing emotional and behavioural vulnerability. For example, the family histories of rheumatic fever in our study were in keeping with known heritability [24]. Family histories of psychiatric disorders are of interest, but the numbers were too small in our study to suggest links to a child's vulnerability to SC. For example, one study found that nearly half of UK adults reported at least one of eight forms of common mental disorders in their lifetime [25] Another stated that maternal reports of depression after the onset of SC may have reflected disease‐related stress, associated with adverse psychological adjustments in caregivers and children [26].

Our finding of persistent signs of chorea in five cases was in keeping with the 40% rates described in a meta‐analysis [1]. The same meta‐analysis found a relapse rate of 34% in reported cases of SC [1]. The rate of 75% in our study was higher, although 25% described relapses with predominant psychiatric symptoms, which may not have been identified as SC relapses in every setting. This finding may have been a result of our retrospective review methodology. It is possible that children with SC, which is complicated by psychiatric symptoms, may have a more difficult course, with varying neuropsychiatric sequelae of SC plus chorea in relapses. Children's persistent or relapsing experiences of the physical and psychiatric aspects of SC support ongoing collaborative management by neurology and psychiatry. The range of drugs that were prescribed in our study further supports a joint approach. For example, studies have identified an increased risk of motor side effects of psychiatric medication for SC [22, 27]. Recurring and persistent psychiatric symptoms after streptococcal infections, in the absence of ongoing chorea, indicate parallels between SC and PANDAS. Proposed diagnostic criteria [26] suggest that PANDAS has a more sudden onset, with children displaying psychiatric symptoms of obsessive‐compulsive disorder and tics. In contrast, the children in our study presented with a mixture of psychiatric symptoms and these tended to show a gradual onset. Chorea was frequently not specifically described as a symptom of past episodes in our study, even in children who were quite severely affected. Some had current signs of chorea, without reporting subjective experiences of chorea during direct questioning. Another study pointed out that diagnoses of SC could be missed, and children could be misdiagnosed with PANDAS if insufficient attention was paid to their history and no specific examinations were carried out [28]. Relapsing and remitting symptoms may complicate the course of SC with psychiatric co‐morbidities. The associated variations in educational difficulties and behavioural problems may present challenges for services. Working diagnoses and watchful waiting may be helpful before confirming diagnoses with lifelong implications, such as ADHD or ASD.

Our study followed published contemporary guidance on managing SC [22]. Children and their parents were provided with a clear explanation of the likely course of the disorder and advice regarding using long‐term penicillin to prevent rheumatic heart disease. Occupational therapy, physiotherapy, support with schooling and medication were offered to decrease the burden of abnormal movements [22]. Our study children presented before the current interest in immuno‐modulatory therapies, such as corticosteroids, intravenous immunoglobulins and plasma exchange. These may have shortened the course of their illnesses and prevented complications [29].

Our study comprised around half of the children diagnosed with SC in the West of Scotland tertiary service during a three‐year period of increased SC presentations. This suggests a regional annual incidence of SC of around two to three per 100 000 children, which was more than 10 times greater than previous studies in the UK and Ireland [6, 7]. Some milder cases may not have been identified and not every child with a diagnosis of SC will have reached specialist services. It is possible that not all children with psychiatric symptoms were referred to neuropsychiatry. Cases may have been atypical, as this cluster of SC presentations was unusual, and its cause was unknown.

### 4.1 Limitations

The limitations included the small study size, which restricted its usefulness, and the fact that we did not have access to the children's medical records to supplement the information from the interviews. We had a 75% response rate, but it is unfortunate that there was little information about the non‐responders. The study design did not allow us to draw any conclusions about the possibility that pre‐existing vulnerabilities may have predisposed the children to SC. Very detailed explorations were limited to the most severe episode and this meant that some pre‐morbid conditions may not have been fully delineated. This limited the details available about the psychiatric symptoms that occurred before the SC diagnosis. The psychiatric diagnoses depended upon the K‐SADS‐PL, which does not include the educational or cognitive reports that contribute to diagnoses in clinical practice. The tool has not been specifically validated for use with co‐morbid neurological disorders. The validity of the K‐SADS‐PL has not been established in retrospectively identifying tics and motor over‐activity in the presence of a movement disorder. This may have posed a particular difficulty in relation to reporting tics prior to an SC diagnosis. Clinicians, parents and children may have trouble in discriminating between tics and choreiform movements, as one case study showed [30]. Rates of psychiatric disorders seen in a highly specialist neuropsychiatry clinic are also poor indicators of their wider prevalence in children with SC.

## 5 Conclusion

This retrospective study looked at the experiences of a clinical sample of Scottish children with SC and their families. The children all had sufficient psychopathology to lead to a neuropsychiatry referral, but there were great variations in the disease course and subsequent outcomes. Residual chorea persisted for some children. Some had much more complex physical presentations, with features of rheumatic fever, such as cardiac complications or joint pain. Others had more complex or severe psychiatric disorders and three were attending mental health services at the time of interview. We found more frequent relapses than cohorts recruited from other settings. Delays in diagnosis added more stress for families. Maternal depression was reported to be linked to the stress of their child's illness. These findings highlight the need for access to swift diagnoses and a range of services that can support a full recovery. Future research could explore the possibility of a predisposition to neuropsychiatric symptoms in SC and the possible excess of psychiatric disorders in affected families. Further studies of the course of psychiatric symptoms in SC may also support a better understanding of the relationship between SC and related concepts, such as PANDAS.

## Author Contributions

**Nadine Mushet:** investigation, funding acquisition, writing – original draft, methodology, validation, writing – review and editing, formal analysis, resources. **Michael Morton:** validation, methodology, conceptualization, writing – review and editing, supervision. **Helen Minnis:** supervision, validation, resources. **Christopher Gillberg:** supervision, validation, visualization, methodology.

## Conflicts of Interest

The authors declare no conflicts of interest.
